# Butyrate Modulates Intestinal Microbiome and Epithelial Function to Attenuate Irinotecan-induced GI Toxicity

**DOI:** 10.21203/rs.3.rs-7436690/v1

**Published:** 2025-08-27

**Authors:** Stanley M. Cheatham, Zayd Rehman, Mahshid Arastonejad, Yuesheng Zhang, David A. Gewirtz, Hamid I. Akbarali, Hisashi Harada, Natalie Luffman, Katarzyna M. Tyc

**Affiliations:** Department of Pharmacology and Toxicology, Virginia Commonwealth University, Richmond, 23298, USA; Department of Oral and Craniofacial Molecular Biology, Virginia Commonwealth University, Richmond, 23298, USA; Department of Biostatistics, Virginia Commonwealth University, Richmond, 23298, USA

## Abstract

Chemotherapy-induced gastrointestinal toxicity is a significant dose-limiting complication for cancer treatment. Disruption of the gastrointestinal (GI) epithelial barrier function by several chemotherapeutic agents results in development of mucositis and diarrhea. Thus, maintaining barrier integrity may be of therapeutic benefit. Recent studies have shown the beneficial effects of the microbial metabolite butyrate, a short chain fatty acid (SCFA), on epithelial barrier integrity. In this current study, we tested the effect of oral butyrate on irinotecan-induced gastrointestinal (GI) toxicity in mice. Irinotecan dose-dependently reduced body weight and increased fecal water content. Nicotine-induced inward currents in ileum myenteric neurons were significantly increased in irinotecan treated mice consistent with enhanced GI motility. Loperamide reduced GI motility of irinotecan treated mice, however tolerance developed with chronic use, consistent with clinical findings of loperamide refractory diarrhea in patients. Oral butyrate improved epithelial permeability, prevented loss in stem cell marker, *lgr5* in colonic crypts and *muc2* expression in ileum. Butyrate also prevented irinotecan-induced increase in β-glucuronidase activity in fecal samples. Irinotecan treatment produced a significant shift in the β diversity of the fecal microbiome that was mitigated by butyrate. The microbial dysbiosis was associated with increases in the mucin degrading bacteria *Akkermansia muciniphilia* and the hydrogen sulfide producing *Desulfovibrio sp10575755* that was reduced with butyrate treatment.

## Introduction

Chemotherapy-induced gastrointestinal toxicities (CIGT) are among the most common and most debilitating adverse effects of cancer treatment. Regimens containing irinotecan (CPT-11), 5-fluorouracil (5-FU), and leucovorin (FOLFIRI), as well as, pelvic radiotherapy have been associated with diarrhea and mucositis in 50–80% of patients^1,2^ These toxicities can be life-threatening when severe diarrhea leads to dehydration and electrolyte imbalances, and mucositis compromises oral intake and predisposes patients to infection. In irinotecan-treated patients, diarrhea ranges from mild (grades 1–2) to severe (grades 3–4), with the latter frequently necessitating hospitalization^34^.

Despite advances in oncology, effective management of CIGT remains elusive. Beyond diminishing quality of life, CIGT often forces dose reductions, treatment delays, or even discontinuation of potentially curative therapy. First-line control relies on the peripherally restricted μ-opioid receptor agonist loperamide (up to 16 mg/day), but many patients develop loperamide-refractory diarrhea. In these cases, intravenous octreotide, while sometimes effective, adds cost, requires frequent clinic visits, and still fails to fully resolve symptoms in a subset of patients^5–7^. To date, little progress has been made in treating refractory CIGT^7–10^.

Irinotecan is converted by hepatic and systemic carboxylesterases (CES) into its active metabolite, SN-38, which is subsequently glucuronidated by UGT1A1 to form SN-38G. Both SN-38 and SN-38G are excreted into the intestinal lumen, where bacterial β-glucuronidases deconjugate SN-38G back to SN-38^11,12^. Accumulation of SN-38 in the gut induces epithelial apoptosis, disrupts cell-cell junctions, and triggers excessive cholinergic activation of enteric neurons, together driving secretory diarrhea and altered motility patterns^13^.

Short-chain fatty acids (SCFAs), principally acetate, propionate, and butyrate, are generated by microbial fermentation of dietary fiber in the cecum and colon. Butyrate exerts anti-inflammatory effects via the Gprotein coupled receptor (GCPR) GPR109a, and the free fatty acid receptors (FFAR2/3) on epithelial and immune cells and serves as a class 1 and 2a histone deacetylase inhibitor (HDACi) to modulate gene transcription^14^. Preclinical studies demonstrate that butyrate inhibits pro-inflammatory cytokine production, strengthens epithelial barrier integrity, and can enhance the efficacy of conventional chemotherapeutics^15–17^. Moreover, dietary interventions that increase colonic SCFA production have been shown to attenuate gut injury in models of colitis and mucositis^17,18^.

There is growing evidence of a strong link between the composition of the gut microbiota and the anti-tumor efficacy of chemotherapeutics, including irinotecan^18,19^. There are multiple mechanisms by which the gut microbiome alters the functional effects of irinotecan, which can include shifts in bacterial species that contain β-glucuronidase activity, alteration in metabolic function that affects tryptophan metabolism, and generation of the short chain fatty acids^20–25^. In this study, we examined the effect of butyrate supplementation on irinotecan induced changes in gastrointestinal toxicity. We found that irinotecan-induced disruption of the gut barrier is associated with shifts in the gut microbiome and enhanced β-glucuronidase activity that is prevented by butyrate.

## Methods

### Animals

All animal experiments were approved by the Institutional Animal Care and Use Committee (IACUC) at Virginia Commonwealth University. Eight-week-old male ICR mice (30–44g) (ENVIGO, Indiana, IN USA) were housed 4–5 per cage under a controlled temperature (22 ± 2C), and 12 h light/dark cycle), with food pellets and water *ad libitum*. All efforts were made to minimize animal suffering and to reduce the number of animals used. Animals were randomly allocated to experimental or control groups. Weight loss of mice treated with irinotecan, did not exceed 20% (24–36 g) at the time of euthanasia. Mice were killed using CO_2_ asphyxiation followed by cervical dislocation. This study is performed in accordance with relevant guidelines and regulations. All methods are reported in accordance with ARRIVE guidelines^26,27^.

### Drugs

Mice were treated with once daily of irinotecan hydrochloride (Apotex corp) at 60, 75, or 100 mg/kg intraperitoneally (i.p) for four consecutive days^26^. This model of irinotecan induced GI toxicity parallels intestinal toxicity observed in humans due to the accumulation of SN-38 in intestinal tissue through enterohepatic circulation^27^. The vehicle solution consisted of 0.9% saline, sorbitol 45 mg/ml and 0.9 mg lactic acid and adjusted to a pH of 3.3–3.8. In studies utilizing loperamide ((Sigma-Aldrich, St. Louis, MO), animals received once daily oral gavage (p.o.) of a loperamide solution consisting of 0.5% carboxymethylcellulose in deionized water on day 5 of treatment paradigm. Animals receiving butyrate were given 200 μL twice daily p.o. of 250 mM solution starting 3 days before administration of irinotecan or control followed by continued administration throughout experimental timeline. Similarly, Mecamylamine (Sigma-Aldrich, St. Louis, MO) was administered at 3 mg/kg/day in 0.9% saline starting on day 5 of irinotecan treatment paradigm. Additionally, Atropine (Sigma-Aldrich, St. Louis, MO) was administered at 5 mg/kg in 0.9% saline starting on day 5 of irinotecan treatment paradigm.

### Diarrhea Scoring

Severity and intensity of diarrhea production was followed daily in all groups according to the scoring criteria outlined in Table 1^28^.

### Fecal water content

Mice were placed in bedding free cages with no access to food or water for a period of one hour. Fecal pellets were collected and weighed at the end of the testing session. Pellets were then dried at 37 °C for 24 hours. Water content was calculated as the percentage of weight lost upon drying, using the formula (wet-dry)/(wet)*100.

### Gastrointestinal Transit

Small intestine transit was measured via an oral gavage consisting of 5% aqueous suspension of charcoal (Sigma-Aldrich, St. Louis, MO) in a 10% gum arabica solution mg/kg. At 30 min after the administration of the charcoal meal, the mice were euthanized by CO_2_ asphyxiation followed by cervical dislocation, and the small intestine from the stomach to the cecum was dissected and placed in cold saline to stop peristalsis. The distance traveled by the leading edge of the charcoal meal was measured relative to the total length of the small intestine. The percentage of intestinal transit for each animal was calculated as percentage transit (charcoal distance)/(small intestinal length)^29^.

### Gastrointestinal Motility Monitoring (GIMM)

A gastrointestinal motility monitoring system (Med associates) was utilized to measure the effect of irinotecan on colonic motility ex-vivo. Briefly, animals were euthanized by CO_2_ asphyxiation followed by cervical dislocation, and colonic tissue isolated in warm Kreb’s buffer (118 mM NaCl, 4.6 mM KCl,1.3 mM NaH_2_PO_4_, 25 mM NaHCO3, 1.2 mM MgSO_4_, 11 mMC_6_H_12_O_6_, 2.5mM CaCl_2_). The lumen was flushed with Krebs and then the ends were tied after the lumen was filled with Krebs buffer. The tissue was placed in an illuminated organ bath continuously perfused with Krebs solution bubbled with carbogen gas (95%O_2_/5%CO_2_)^30^. The preparation was allowed to equilibrate for 1h, and then perfused with 1 μM Acetylcholine (Ach). Spatiotemporal maps were video recorded and graphed in (GIMM Software, Med Associates).

### Intestinal Permeability Assay

*In vivo* intestinal permeability was performed following previous work from our lab^31,32^. Briefly, 4kDA FITC-conjugated dextran (Sigma-Aldrich, St. Louis, MO) was dissolved in 1xPBS and administered by oral gavage (44 mg/100 g body weight) 4 hours before whole blood collection by cardiac puncture under isoflurane anesthesia. Plasma was isolated from blood samples by centrifugation for 15 min at 3000 rpm and 4°C. Plasma sample were diluted with an equal volume of 1x PBS. 100μl of samples were then placed in a VWR 96-well plate and FITC concentration was fluorometrically quantified by emission spectrometry (Tecan infinite 200 pro) at Ex/Em = 528/485. All concentrations were measured against a standard curve of serially diluted FITC-dextran (0, 125, 250, 500, 1000, 2000, 4000, 6000, and 8000 ng/ml).

### Histological evaluation

Tissues were fixed with 4% paraformaldehyde, embedded in paraffin, and sliced at 8 μm thickness. Hematoxylin and eosin (H&E) staining were carried out by *VCU Massey Comprehensive Cancer Center Tissue and Data Acquisition and Analysis Shared Resource*

### Assessment of B-glucuronidase activity

On the sixth day following initial administration, mice were individually placed into sanitized, beddingfree cages. Fecal samples were immediately weighed and flash-frozen in Eppendorf tubes to preserve the original bacterial composition. These samples were then stored at −80°C in a freezer for later analysis. The β-glucuronidase activity was determined using the β-Glucuronidase Activity Assay Kit (Fluorometric) (ab234625; Abcam) according to the manufacturer’s instructions. Briefly, samples were initially weighed and placed on ice, followed by the addition of Assay Buffer at a ratio of 100μl per 10 mg of sample. Samples were homogenized twice using sonic homogenizers, then centrifuged at 10,000g for 5 minutes at 4°C to collect the supernatant, which was kept on ice. Positive controls were reconstituted with 55μl of Assay Buffer, while standards were prepared by mixing 72μl of Assay Buffer with 3μl of Standard stock solution. The substrate was diluted 10-fold with Assay Buffer. Using a multi-well plate compatible with the TECAN Infinite 200 Pro spectrophotometer, each sample was pipetted into duplicate wells with 88 or 89μl of Assay Buffer and 2 or 1μl of sample supernatant, respectively, and 10μl of substrate. To ensure the experimental data fit within the standard curve, the volume of the sample supernatant and assay buffer was adjusted as necessary. Sample blanks (90μl Assay Buffer + 10μl substrate) and blanks (100μl Assay Buffer) were included. A standard curve was generated with wells containing duplicate volumes of Assay Buffer (98, 96, 94, 92, 90μl) and known volumes of 10X standard solution (2, 4, 6, 8, 10μl). Fluorescent data was collected in 5-minute cycles over the course of an hour at 37°C. Fluorescence was measured immediately after substrate addition at excitation and emission wavelengths of 330nm and 450nm, respectively, while temperature was held at 37°C. This was continued for cycles of 5 minutes over the course of an hour. Each fluorescent reading was averaged with its duplicate, followed by subtracting the fluorescence of the Blank to each well. The remaining fluorescence values in RFU were converted to 4-MUnmol using the standard curve. Using Eq. 1 and the respective 4-MUnmol values, activity was expressed as μU/mg.


(1) $$\mu \text{U}/\text{mg}=\left(\text{B}2-\text{B}1\right)/\left(\text{t}*\text{V}\right)$$

Where:

B2 = activity of sample at time2

B1 = activity of sample at time1

t = time2 - time1

V = volume of sample in mL

### qPCR

Reverse-transcriptase polymerase chain reaction (RT-PCR) was performed on a Mini-Opticon real-time PCR system (Bio-Rad, Hercules, CA). Glyceraldehyde-3-phosphate dehydrogenase (GAPDH) was used as an internal control. Sample tissues were isolated, and total RNA was extracted using TRIzol reagent (ThermoFisher Scientific, Waltham, MA). DNase I (New England Biolabs, Ipswitch, MA) was used to remove any residual contaminating DNA. Relative expression of the respective genes to GAPDH expression was calculated using the ΔΔCt method and values were expressed as fold change from respective controls.

Primers were purchased from Integrated DNA Technologies (IDT) (table 2)

### Shotgun sequencing of fecal microbiome

Fecal samples were collected from irinotecan, vehicle, irinotecan + butyrate, and vehicle + butyrate treated mice. Microbial DNA was isolated from the fecal samples using the QIAamp PowerFecal Pro DNA Kit (QIAGEN, Aarhus, Denmark) according to the manufacturer’s protocols, and subjected to whole genome shotgun sequencing using the Nextseq 2000 platform from Illumina by VCU Genomics Core facility.

Demultiplex FASTQ files were uploaded to CosmosID (www.cosmosidhub.com) and bioinformatic analysis was executed according to proprietary methods on the raw data to generate taxonomic and relative abundance estimates.

### Enteric Neuron Isolation

Ileum isolation proceeded as previously outlined in Smith et. al.^33^. Briefly, the ileum was immediately dissected and placed in ice-cold Krebs solution (in mM: 118 NaCl, 4.6 KCl, 1.3 NaH_2_PO_4_, 1.2 MgSO_4_ 25 NaHCO_3_, 11 glucose and 2.5 CaCl_2_) bubbled with carbogen (95% O_2_/5% CO_2_). Ileal segments were threaded longitudinally on a plastic rod through the lumen and the longitudinal muscle with the myenteric plexus (LMMP) was gently removed using a cotton-tipped applicator. LMMP strips were then minced with scissors and digested in 1.3 mg/ml collagenase type II (Worthington) and 0.3 mg/ml bovine serum albumin (BSA) at 37°C for 1 hour. Following digestion, cells were triturated and collected by centrifuge (350× *g* for 8 min) followed by 0.05% trypsin in Hank’s balanced buffer solution (HBSS) for 7 min. Cells were then plated on laminin (BD Biosciences) and poly-D-lysine coated coverslips in Neurobasal A media containing B-27 supplement, 1% fetal bovine serum, 10 ng/ml glial cell line-derived neurotrophic factor (GDNF, Neuromics, Edina, MN), and antibiotic/antimycotic liquid. Cell media was changed every 2–3 days.

### Electrophysiology

Standard whole-cell configuration was used for all recordings as reported previously by our lab in Smith et. al. and Gade et.al.^34,35^. An EPC 10 amplifier (HEKA, Bellmore, NY) was used for recordings. All patch-clamp recordings were performed in enteric neurons within 2 days after isolation. Coverslips with attached cells were placed in a recording chamber under an inverted microscope and continuously perfused with external solution containing the following (in mM): 135 NaCl, 5.4 KCl, 0.3 NaH_2_PO_4_, 1 MgCl_2_, 5 glucose, and 2 CaCl_2_ (pH adjusted to 7.4 using 1 M NaOH). The patch pipettes were prepared using a Flaming-Brown horizontal micropipette puller (P-87; Sutter Instrument, Novato, CA) and fire polished. Resistance of the pipettes used was 1.5–2.5 MΩ when filled with an internal solution containing the following (in mM): 100 K-aspartic acid, 30 KCl, 4.5 ATP, 1 MgCl_2_, 10 HEPES, and 0.1 EGTA. Series resistance was < 10 MΩ and not compensated. The currents were measured by using a gap-free protocol at − 70 mV. Nicotine was applied to the cell via bath perfusion.

### WST-1 Assay

MDA-MB-231 cells (10,000 cells per well) were seeded into a 96-well plate and allowed to adhere overnight. SN-38 was administered at concentrations of 0, 1, 2, 5, 10, 20, 50, and 100 μM +/− 10 μM sodium butyrate and allowed to incubate for 72 hours. WST-1 reagent was allowed to incubate for 4 hours, and absorbance was measured at 450 nm on a Promega Glomax spectrophotometer.

### Statistical Analysis

Statistical analyses were performed with GraphPad 10.5.0 software (GraphPad Software Inc., San Diego, CA, USA). Data are presented as means ± SEM, with data points from individual animals (n). Statistical analysis was undertaken using individual replicate values, when sample sizes were at least n = 4–5. One-way analysis of variance (ANOVA) was used for statistical evaluation of differences between groups (n > 2) with Bonferroni’s post hoc. 2 way-ANOVA for repeated measures with Tukey’s post hoc test was used for temporal comparisons in any given group. Repeated measures ANOVA was used for continued temporal assessment of CIGT. IC50 and 80 (Y = Bottom + (Top-Bottom)/(1+(X/IC50) values were calculated for loperamide inhibition of gastrointestinal transit. T-tests were used to compare differences between two paired or unpaired groups.

### Data availability

Raw data for mouse metagenomics are publicly available via sequence read archives (SRA) under the Bioproject accession ID PRJNA1300309.

## Results

### Gastrointestinal toxicity during irinotecan treatment

**Figure 1:**
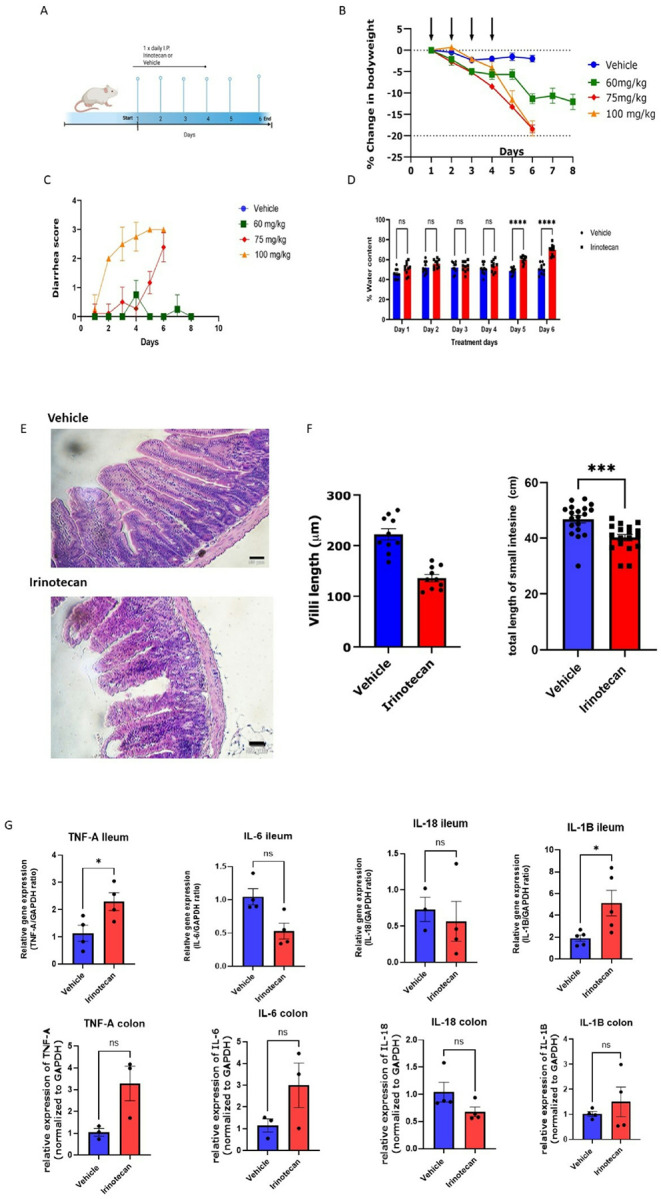
Model of irinotecan induced gastrointestinal toxicity. **(A) T**reatment timeline. **(B)** body weight is reduced over the treatment period of irinotecan. **(C)** Diarrhea score increases during irinotecan treatment period. repeated measures One-way ANOVA vehicle n=10, irinotecan n=10. **(D)**Fecal water content increases over six days of the irinotecan treatment (vehicle n= 10, irinotecan n= 10 repeated measures ANOVA** P<0.01. **(E)** H&E staining of ileum sections from vehicle and irinotecan treatment revealed a blunting of tips and reduction in villi length as well as marked atrophy of crypt and villi architecture (vehicle n=10, irinotecan n=10. **(F)** small intestine length is decreased in the irinotecan treatment group n=19 compared to vehicle n=19 un-paired t-test **** p<0.001, but colon length remains unaffected. (**G)** qRT-PCR of TNF-a, IL-1β, and IL-6 from isolated ileum on day 6 of treatment (vehicle n=4–5 irinotecan n=4–5, un-paired t-test * p<0.05)

Irinotecan or vehicle was administered to mice once daily via i.p. at either 60, 75, or 100 mg/kg for a period of four days (Fig. 1A). Animals were assessed for body weight, diarrhea, and fecal water content daily until reaching a maximal 20% reduction in body weight as per IACUC guidelines. Animals treated with 60 mg/kg irinotecan lost body weight but did not present with any qualitative assessment of diarrhea (Fig. 1B, C). Mice treated with 75 or 100 mg/kg irinotecan presented with significant body weight loss, severe diarrhea as measured through diarrheal assessment scale (Fig. 1C), and increased fecal water content (Fig. 1D). Treatment with 100 mg/kg/day irinotecan proved to be too severe in nature, and thus 75 mg/kg was chosen as the test dose of irinotecan. These findings are similar to previous studies where 75 mg/kg irinotecan presented with progressive reduction of body weight, achieving a 20% reduction by day 6^16,17,36–38^. For all future studies of irinotecan induced gastrointestinal toxicity, a once daily dose of 75 mg/kg for 4 consecutive days was selected.

Marked atrophy and disruption of crypt architecture was evident in H&E-stained cross-section of the ileum of irinotecan treated mice with significant shortening of villi length (Fig. 1E). Furthermore, the total small intestine length was significantly shorter as compared to their vehicle controls following irinotecan treatment (Fig. 1F). However, there was no change in length of colons (not shown). The reduction in small intestine length is consistent with inflammation-induced shortening in models of experimentally induced colitis (Tri-nitro-Benzene Sulfate (TNBS) and Dextran Sodium Sulfate (DSS)^39^.

We next determined the expression of pro-inflammatory cytokines in the ileum and colon of irinotecan-treated mice. There was significant increase in both TNF-α and IL-1β in the ileum, whilst in the colon, TNF-α and IL-6 were increased, but not significant (Fig. 1G). These data are consistent with irinotecan inducing significant toxicity in the gastrointestinal tract.

### Gastrointestinal motility

**Figure 2:**
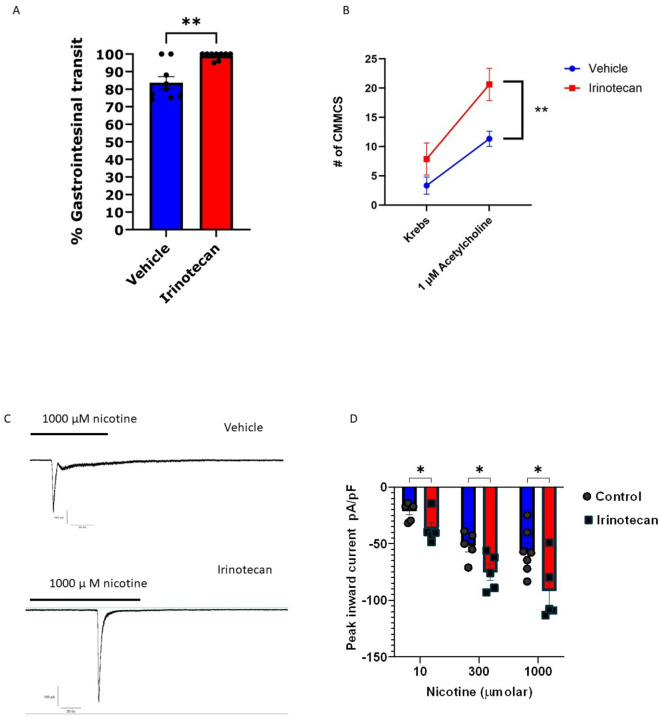
Altered gastrointestinal motility is associated with increased cholinergic tone. **(A)** Small intestine transit is enhanced during irinotecan treatment un-paired t-test *p<0.05 (**B)** *Ex-vivo* stimulation of colonic tissues with 1 uM ACh significantly increases CMMCs from baseline. (Irinotecan n=9, vehicle n=8, two-way ANOVA, *p<0.01). (**C)** Representative traces of nicotine administration to vehicle and irinotecan treated myenteric neurons. **(D)** Dose dependent increase in inward currents from isolated myenteric neurons on day 6 of irinotecan treatment upon perfusion with 10,300, or 1000 μM nicotine (irinotecan n=5, vehicle n=5–6,un-paired t-test, *p<0.05).

To determine whether gastrointestinal motility is altered by irinotecan-treatment, small intestinal transit was determined by measuring the distance traveled by oral charcoal gavage. The distance of the charcoal travel was measured from pyloric sphincter to ileocecal end in vehicle and irinotecan-treated mice. In 30 minutes, the charcoal traversed the small intestine to 80% of its length in control animals while entering the colon in irinotecan-treated mice (Fig. 2A), indicative of enhanced transit.

We next tested whether irinotecan treatment enhances smooth muscle contractions. For these studies, colonic migrating motor complexes (CMMCs) were measured in *ex-vivo* preparations of colonic tissue from irinotecan and vehicle-treated animals by spatiotemporal mapping using the GIMM system.^40^ The number of CMMCs were measured over a 30 min period in Krebs solution followed by 30 min in response to acetylcholine (1 μM) (Supplement Fig. 1). Under basal conditions, the number of CMMCs were slightly higher in colons from irinotecan-treated mice (7 ± 2; n = 8) than in vehicles (3 ± 1; n = 9). While the basal contractions did not reach significance, addition of acetylcholine markedly enhanced the contractions in the colons from irinotecan-treated mice (20 ± 2; n = 8) compared to vehicle-treated colons (11 ± 1, n = 8) (Fig. 2B).

We further tested whether cholinergic neuronal activation was affected by irinotecan treatment. For this purpose, we conducted voltage clamp studies in isolated ileum myenteric neurons^41^. Cells were held at −70 mV and perfused with nicotine. As noted previously^42^ nicotine induced dose-dependent inward currents in myenteric neurons. The peak inward currents (pA/pF) were significantly greater in neurons from irinotecan-treated mice at 10 μM (−37.06 ± 5.89 vs −21 ± 3.04 SEM), 300 μM (−75.2 ± 7.4 vs −51.54 ± 5.6 SEM), and 1000 μM (−91.76 ± 12.2 vs −57.0 ± 7.4 SEM) nicotine when compared to myenteric neurons from vehicle-treated mice (Fig. 2C, D). These data suggest that the cholinergic activation is enhanced by irinotecan treatment. We then determined whether treatment with the nicotinic antagonist affects irinotecan-induced toxicity. Mecamylamine (3 mg/kg) or Atropine (5mg/kg) treatment following irinotecan improved body weight loss indicative of cholinergic modulation of gastrointestinal toxicity (Fig S2).

### Therapeutic efficacy of loperamide in irinotecan-induced CIGT

**Figure 3:**
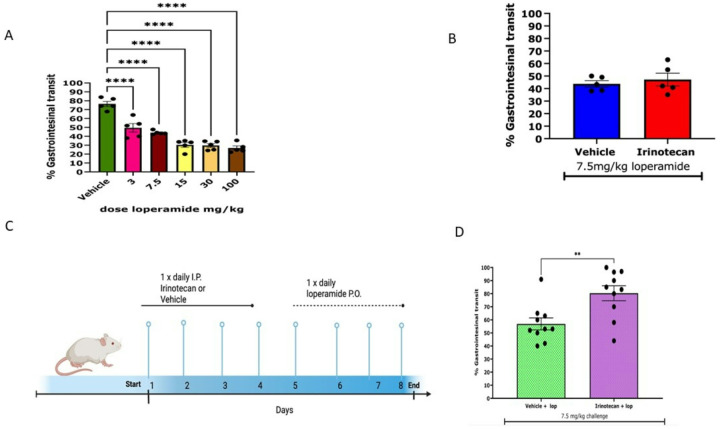
Loperamide inhibits gastrointestinal transit acutely but not chronically in a dose dependent manner. **(A)** Loperamide exhibits a dose dependent decrease in gastrointestinal transit. There is a significant reduction in transit at 3 mg/kg,7.5 mg/kg, 15 mg/kg, 30 mg/kg and 100 mg/kg relative to vehicle solution. IC50 and 90 values were 3.12 and 28.37 respectively. **(B)** Acute administration of 7.5 mg/kg loperamide on day six of irinotecan treatment reduces gastrointestinal motility (vehicle n=5 irinotecan n=5 unpaired t-test * p<0.05). **(C)** Repeated administration of loperamide**(F)** Motility becomes tolerant to decreases in gastrointestinal motility.

The peripheral opioid agonist, loperamide, is used as first line therapy for CID. To determine the effect of loperamide on irinotecan-induced gastrointestinal dysfunction, we first conduced a dose-response experiment for inhibition of gastrointestinal motility by oral loperamide. The distance traveled by charcoal from the pylorus to the cecum was determined as the percent gastrointestinal transit. Figure 3A shows that loperamide exhibited a dose-dependent reduction in transit in naïve mice, with 3 mg/kg reducing small intestinal transit to 49% of total length, 7.5 mg/kg reducing transit to 44%, 15 mg/kg reducing transit to 30.42%, 30 mg/kg reducing transit relative to 29%, and 100 mg/kg reducing transit to 26.19% of the distance with an IC50 of 3.12 mg/kg and IC90 of 28.37 mg/kg. To investigate if an acute dose of loperamide was efficacious in reducing gastrointestinal transit during irinotecan treatment, animals received an oral dose of 7.5 mg/kg loperamide on day 6 of irinotecan treatment. This dose of loperamide reduced gastrointestinal motility in irinotecan treated mice to the same extent as in vehicle-treated mice (Fig. 3B), indicating that an acute dose of loperamide was equally efficacious in reducing gastrointestinal motility following chemotherapy.

To determine whether loperamide prevents gastrointestinal toxicity, we measured weight loss in mice that were treated with irinotecan for 4 days, followed by daily treatment with loperamide (15 mg/kg) (Fig. 3C). At this dose of loperamide, the 20% reduction in weight loss was observed at day 8 post irinotecan treatment compared to irinotecan alone (Fig S3). This suggests that while loperamide treatment delays the onset of weight loss, it nevertheless only prolongs the time to reach the weight loss cutoff. This is consistent with clinical findings of loperamide-refractory GI toxicity^7^. We therefore tested whether tolerance occurs to loperamide-induced inhibition of gastrointestinal motility and diarrhea. For these studies, we determined the effect of a challenge dose of loperamide to reduce small intestinal transit in irinotecan-treated mice that also received loperamide for 4 days. A 7.5 mg/kg p.o. challenge dose of loperamide reduced transit in vehicle-treated mice to 55 ± 5% compared to only 80 ± 6% of the small intestinal length in the irinotecan-treated mice (Fig. 3D). This reduced inhibition in the irinotecan-treated mice that received daily loperamide is indicative of enhanced tolerance to irinotecan.

### Butyrate restricts the development of irinotecan-induced gastrointestinal toxicity

**Figure 4:**
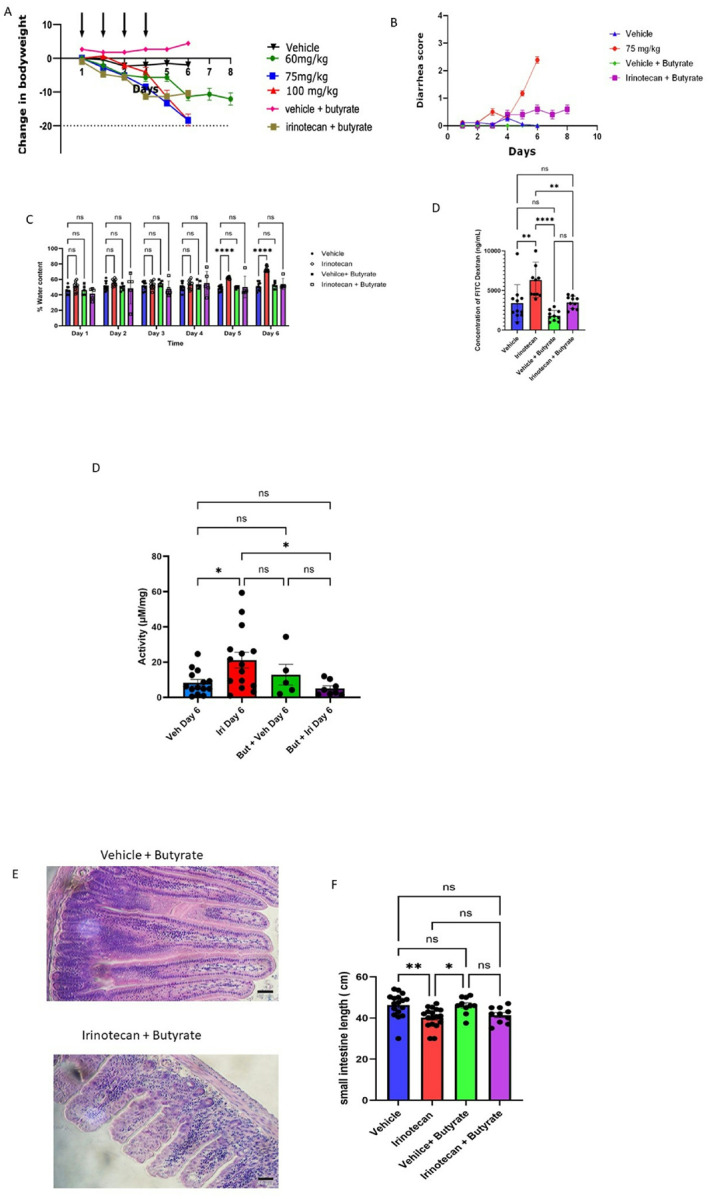
Butyrate treatment ameliorates irinotecan induced gastrointestinal toxicity **A)** Butyrate treatment improves weight loss **(B)** Increases in diarrhea score are attenuated by butyrate. **(C)** Fecal water content in butyrate treatment groups is decreased; repeated-measures ANOVA. **(D)** Barrier integrity is improved by butyrate in 4kDa FITC-Dextran assay, one-way ANOVA, *p<0.05. **(E)** Representative H&E staining of vehicle+ butyrate, and irinotecan + butyrate treated ileum. **(F)** Small intestine length is not improved by butyrate treatment. vehicle + butyrate n=5, irinotecan butyrate n=5)

We next determined whether improving barrier integrity reduces irinotecan-induced GI toxicity. Butyrate is a short chain fatty acid that improves epithelial barrier integrity^16,36,37,43^. We began daily butyrate treatment 200μL (250 mM p.o. b.i.d.) 3 days prior to irinotecan or vehicle treatment^44^. Oral butyrate significantly improved weight loss (Fig. 4A), diarrhea score (Fig. 4B), and fecal water content on day 5 and 6 post-irinotecan treatment (Fig. 4C). In order to confirm that butyrate improves epithelial barrier integrity, *in-vivo* permeability assays were performed to investigate changes in epithelial permeability. Barrier integrity was assessed by oral administration of 4kDa FITC-dextran. Compared to vehicle controls, irinotecan-treated mice exhibited a greater serum concentration of 4kDa FITC-dextran. The concurrent administration of butyrate to irinotecan-treated mice significantly reduced detection of 4 kDa FITC-dextran, indicating an improvement in epithelial barrier integrity (Fig. 4D). The addition of butyrate in vehicle treated animals demonstrated a trend toward a decrease of serum concentration but did not differ from non-butyrate treated vehicle controls. In addition to improving permeability, butyrate also improved the histological assessment of ileal sections (Fig. 4E) but did not improve reduction in small intestinal length (Fig. 4F).

### Butyrate treatment restores Lgr5 and Muc2 expression in the colon and ileum

**Figure 5:**
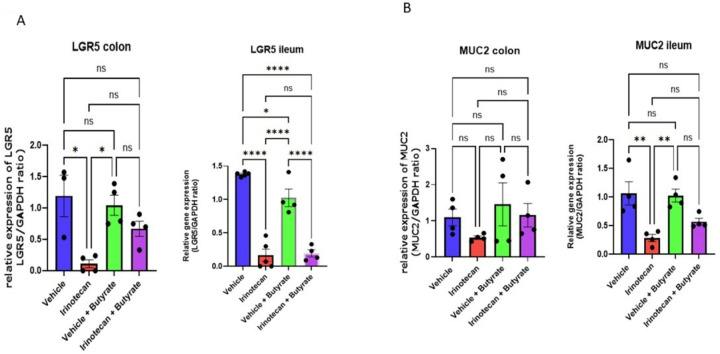
*Muc2* and *Lgr5* expression are restored by butyrate treatment **(A)** and **(B)** qRT-PCR of ileum and colon on day 6 of treatment (vehicle n=4–5, irinotecan n=4–5, vehicle +butyrate =4–5, irinotecan +butyrate n=4–5, one-way ANOVA * p<0.05)

Intestinal stem cells located in the crypts allow for the rapid turnover and differentiation to various cell types in the epithelium. The stem cell marker leucine-rich-repeat-containing G-protein-coupled receptor 5 (Lgr5) is critical in the maintenance of epithelial barrier integrity by allowing efficient tissue repair. Irinotecan significantly reduced expression of Lgr5 in the colon and ileum following irinotecan treatment (Fig. 5A). The decrease in Lgr5 during irinotecan treatment was ameliorated with co-administration of butyrate in the colon, but not the ileum.

We also determined the expression of Mucin-2 (Muc-2), which is a major contributor to the mucosal barrier in intestinal epithelium, providing a protective layer between gut luminal contents and direct interaction with intestinal epithelial cells. Irinotecan treatment did not produce a statistically significant reduction in Muc-2 expression in the colon, although the reduction of Muc-2 in the ileum was significant; however, this was not prevented by butyrate (Fig. 5B). These results suggest that irinotecan alters the expression of both stem cells and mucin production, which may, however, be differentially affected by butyrate in the ileum and colon.

### Butyrate reduces Beta-Glucuronidase activity during irinotecan treatment

**Figure 6:**
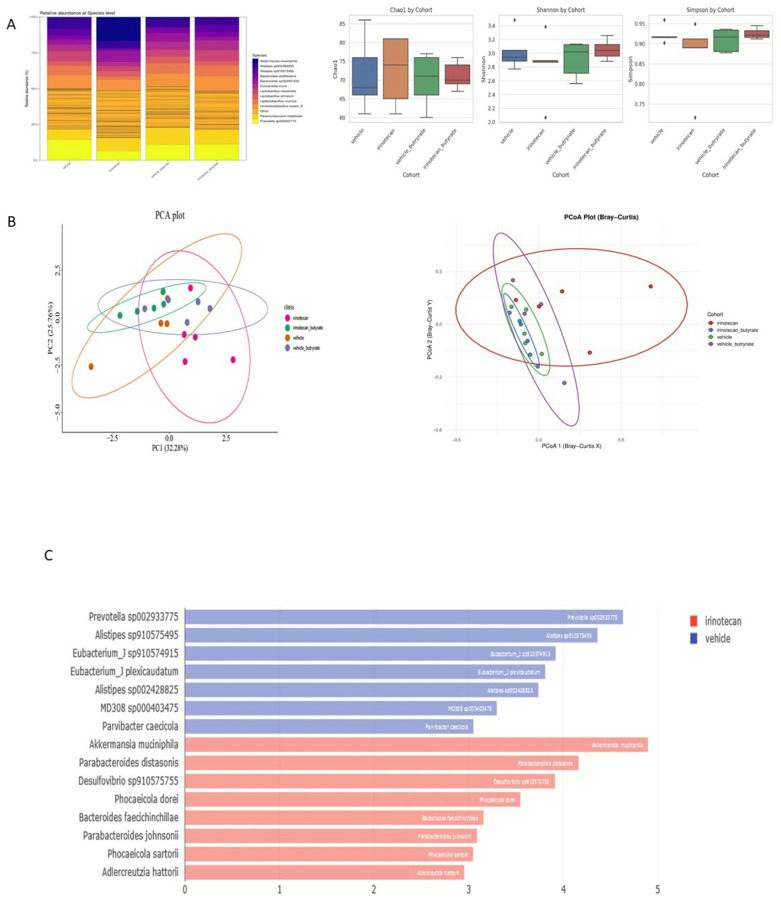
Butyrate modulates the fecal microbiome. **(A)** Mice treated with vehicle, irinotecan, vehicle+butyrate, or irinotecan+butyrate show differences in microbiota composition at the species level. Data points represent averages. Alpha diversity across treatment groups reveals no significant change in species richness across all groups, linear regression on observed species. (**B)** Beta diversity across treatment groups. Microbial compositions differ between vehicle and irinotecan treatment, in addition to, irinotecan and irinotecan + butyrate treatment groups (Bray-Curtis, PERMANOVA with 999 permutations, P<0.05). (**C)** Taxonomic evaluation of microbiota in treatment groups (n=5 mice per group). LeFse analysis highlights strain level differences between vehicle and irinotecan treatment groups.

Gastrointestinal toxicity produced by irinotecan has been attributed to a population of bacteria in the gastrointestinal tract that produce the enzyme beta-glucuronidase^45,46^. Conversion of glucuronidated SN-38G to the active metabolite, SN-38, by the commensal bacteria results in enhanced GI toxicity^47^. On day 6 of treatment, fecal samples were isolated from vehicle, irinotecan, vehicle + butyrate, and irinotecan + butyrate groups. In response to irinotecan treatment there is a significant increase in beta-glucuronidase activity (Fig. 6A), which was prevented in the presence of butyrate. These results suggest that treatment with irinotecan may independently alter the gut microbiome in such a way that beta-glucuronidase producing bacteria are increased, which would further enhance gastrointestinal toxicity due to SN-38.

### Butyrate alters the composition of fecal microbiota during irinotecan treatment

To assess the impact of irinotecan and butyrate on the gut microbiome, fecal samples were collected on day 6 following the initiation of treatment. Shotgun metagenomic sequencing was conducted to evaluate drug-induced alterations in microbiota composition. There were distinct changes in bacterial composition among the various treatment groups. Evaluation of the alpha diversity (within group diversity) of the fecal bacterial communities revealed no differences in the species richness (Chao1 index), in diversity (Shannon index), or in evenness (Simpson index) in each cohort (Fig. 6A). Principal component analysis (PCA) revealed distinct clustering of bacterial populations based on treatment. However, β-diversity analysis using principal coordinates analysis (PCoA) of Bray-Curtis dissimilarity demonstrated significant differences in microbial community structure between the vehicle and irinotecan (p = 0.02) and irinotecan to irinotecan + butyrate (p = 0.004) groups, indicative of changes in the gut microbiome induced by irinotecan (Fig. 6B).

Linear Discriminant Analysis Effect Size (LEfSe) comparison between irinotecan- and vehicle-treated mice identified a significant increase of *Akkermansia* and *Desulfovibrio* species in the irinotecan cohort (Fig. 6C). Interestingly, *Akkermansia Muciniphila*, a mucin-degrading bacterium, is known to support mucosal barrier integrity and modulate host-microbiota interactions, however there is evidence that may suggest context dependent benefit^48–51^. Its increased abundance may represent a compensatory response to epithelial damage or altered mucin turnover induced by irinotecan treatment. Conversely, the rise in *Desulfovibrio, a sulfate-reducing* bacterium known to generate pro-inflammatory sulfur compounds may contribute to local inflammation and exacerbate mucosal injury.

Taken together, these findings highlight irinotecan’s ability to remodel the gut microbial landscape and underscore the need for further mechanistic studies to elucidate how such changes influence mucosal barrier function and gastrointestinal toxicity.

### Butyrate treatment does not impact SN-38-induced cell death

Butyrate has been previously shown to exert a paradoxical effect on cell growth supporting homeostasis and proliferation in normal epithelial cells while inhibiting aberrant growth and inducing apoptosis in cancer cells. This duality is thought to arise, in part, from differences in cellular metabolism. In cancer cells, the Warburg effect characterized by a preference for aerobic glycolysis over oxidative phosphorylation alters butyrate metabolism, allowing it to accumulate in cells and function as a histone deacetylase inhibitor (HDACi), thereby modulating gene expression and promoting cell cycle arrest or apoptosis^52^.

**Figure 7:**
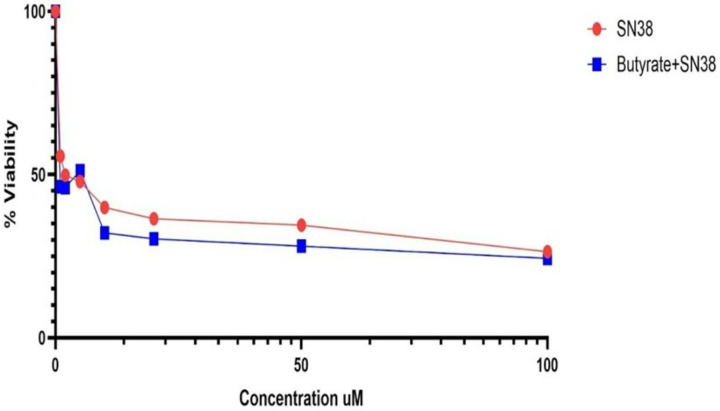
Butyrate does not modulate SN-38 induced cell death in MDA-MB-231 cells. MDA-MB-231 cells treated with SN-38 or SN-38 + 10 μM butyrate demonstrated no impact in cell death. (~10,000 cells per well, three replicates)

To investigate whether butyrate enhances the cytotoxic effects of SN-38, we treated MDA-MB-231 breast cancer cells with SN-38 alone or in combination with 10 μM butyrate. Our results show that co-treatment with SN-38 and butyrate did not result in a significant increase in cell death compared to SN-38 alone (Fig. 7). This finding suggests that, under the tested conditions, butyrate does not potentiate nor hinder SN-38-induced cytotoxicity in MDA-MB-231 cells.

## Discussion

In this study, we show that butyrate supplementation significantly mitigates irinotecan induced gastrointestinal toxicity in mice, as evidenced by sustained body weight, lower diarrhea scores, and reduced histopathological injury. Changes in body weight and inflammatory markers closely mirror those reported in other preclinical models of irinotecan-induced gastrointestinal toxicity. For example, Gibson et al. demonstrated significant weight loss, and small intestine shortening during irinotecan treatment^53^. Similarly, Xue et al. reported comparable inflammatory profiles and histological damage in tumor bearing rats, supporting the robustness of this model.

Gastrointestinal motility is regulated by enteric neural circuity that control circular and longitudinal smooth muscle to drive peristalsis. The primary excitatory neurotransmitter involved in the propagation of contraction includes acetylcholine that acts on nicotinic acetylcholine receptors found on neurons and muscarinic acetylcholine receptors on muscle^54^. We observed a pronounced increase in myenteric neuronal excitability following chemotherapy treatment that was associated with an inflammatory phenotype. This aligns with earlier findings that TNBS-induced colitis enhances enteric neuron firing frequency^55^. McQuade and colleagues similarly reported upregulation of colonic contractions and the proportion of choline acetyl transferase (CHAT) positive neurons in colonic tissues^56^, consistent with the enhanced cholinergic contraction recorded in GIMM experiments.

Although loperamide, a peripherally restricted μ-opioid receptor agonist, remains the first-line therapy for CIGT, its efficacy is limited. A subset of patients still progresses to severe (grade 3–4 diarrhea despite high-dose treatments. Prolonged opioid exposure independently induces gastrointestinal inflammation, and in colitis models we have shown that opioid analgesic tolerance develops more rapidly^59^. Given that irinotecan treatment also elevates proinflammatory cytokine levels, we hypothesized that using loperamide to manage CIGT could enhance the development of tolerance to gastrointestinal transit. We found that while the concurrent administration of loperamide provided modest relief to the loss in body weight, tolerance to the reductions in gastrointestinal transit develops rapidly, which aligns with the clinical findings that many patients become refractory to loperamide despite high doses. In pharmacological evaluations, loperamide effectively slowed gastrointestinal transit and reduced diarrhea severity in our mice, paralleling its established first line use in clinical irinotecan regimens^60^. However, clinical studies indicate that up to 30% of patients experience loperamide-refractory diarrhea, necessitating second-line agents such as octreotide or anticholinergics^6,7^. These limitations underscore the need for adjunctive strategies to improve patient outcomes.

Irinotecan is converted to its active metabolite SN-38, which is glucuronidated by UGT1A1 to SN-38G in the liver and excreted into the gut lumen. There, bacterial β-glucuronidases convert SN-38G back to SN-38, prolonging exposure^59^. We observed that irinotecan alone significantly altered microbial composition, and elevated fecal β-glucuronidase activity, creating a feed-forward loop in which irinotecan treatment alters composition in such a way as to amplify its toxic side effects^60,61^. Butyrate supplementation prevented these microbial shifts, suppressed β-glucuronidase activity, maintained barrier integrity, and Lgr5 stem-cell populations. The reduction in β-glucuronidase limits the reactivation of SN-38G to the toxic SN-38 metabolite. This finding dovetails with studies showing that selective β-glucuronidase inhibitors can attenuate irinotecan toxicity in rodent models without diminishing effects on tumor progression^62,63^. Together, these data reveal that butyrate protects against chemotherapy-induced gut injury by both microbiota-dependent and -independent mechanisms.

The intestinal epithelium and mucosal barrier prevent luminal bacteria from colonizing underlying tissue or translocating into the systemic circulation by deploying multiple, complementary defenses that preserve host microbiota homeostasis. Microbiome profiling revealed that irinotecan treatment markedly altered community beta diversity, shifting away from baseline composition, similar to the findings reported previously^58^. In particular, shotgun sequencing of the fecal microbiome revealed increases in the mucin degrading bacteria *Akkermansia Muciniphila* which has been reported to be beneficial in many contexts including diabetes and obesity^64^. However, many conflicting results have been reported about the role of *A. Munciphilia* on gastrointestinal related inflammation. It has been demonstrated that *A.Munciphilia* may exert contextual and strain dependent anti-inflammatory effects through modulation of mucosal turnover^65^. Recent studies found that levels of *A. Munciphilia* are increased in chronic morphine treated mice that demonstrate compulsive behavior, an indication of a pathobiont^31^.

Desulfovibrio species are Gram-negative, obligate anaerobes that use sulfate as a terminal electron acceptor, producing hydrogen sulfide (H2S) as a metabolic byproduct. Elevated levels of these sulfate-reducing bacteria have been observed in patients with inflammatory bowel diseases including ulcerative colitis and Crohn’s disease, where H2S is thought to exacerbate mucosal inflammation by disrupting epithelial barrier integrity and inhibiting mitochondrial respiration in colonocytes^66–68^. As a representative sulfate-reducing genus, Desulfovibrio’s capacity for H2S generation underscores its potential role in the pathogenesis and maintenance of chronic intestinal inflammation^46–48^. Butyrate co-treatment partially reversed these changes, notably the significant increase in *Desulfovibrio* and *Akkermansia munciphilia*.

Butyrate is particularly interesting due to its context-dependent effects: it supports proliferation and repair in normal intestinal epithelial cells yet exerts anti-proliferative and pro-apoptotic effects in cancer cells.^69^ Given its anti-inflammatory properties, its ability to reinforce barrier function, and its metabolic specificity for normal versus cancerous cells, butyrate holds strong potential as a prophylactic or adjunctive treatment for CIGT.

Collectively, these results demonstrate the direct effects of irinotecan on gut toxicity and highlights butyrate’s multifaceted protective effects modulating immune responses, neuronal function, microbial metabolism, and epithelial integrity to mitigate irinotecan-induced gastrointestinal toxicity. Our studies further suggest that preserving epithelial barrier function may mitigate the significant irinotecan-induced GI dysfunction, a limiting factor in chemotherapy. Future studies should explore combination approaches pairing butyrate or β-glucuronidase inhibitors with standard antidiarrheals to optimize therapeutic efficacy in clinical settings.

## Supplementary Material

This is a list of supplementary files associated with this preprint. Click to download.

- SupplementalFigurelegend.docx

## Figures and Tables

**Table:** 

Loose watery stools, severe perianal staining of coat	3
Moderate wet, unformed, perianal staining	2
Slightly wet and soft	1
No diarrhea	0

**Table:** 

*Gapdh*	Forward: 3’ ggtgaaggtcggtgtgaacgga5’ Reverse: 5’ tgttagtggggtctcgctcctg 3’
*Muc2*	Forward: 3’gctgacgagtggttggtgaatg5’ Reverse: 5’ gatgaggtggcagacaggagac 3’
*Lgr5*	Forward: 3’ ccaatggaataaagacgacggcaaca 5’ Reverse: 5’ gggccttcaggtcttcctcaaagtca3’
Tnf-α	Forward: 3’gttgtaccttgtctactccc5’ Reverse: 5’gtatatgggctcataccagg3’
*Il-6*	Forward: 3’tac cacttcacaagtcgg aggc5’ Reverse: 5’ctgcaagtgcatcgttgttc3’
Il-1β	Forward: 3’ cccaactggtacatcagcac5’ Reverse: 5’ tctgctcattcacgaaaagg3’
